# Loss of ARHGAP15 affects the directional control of migrating interneurons in the embryonic cortex and increases susceptibility to epilepsy

**DOI:** 10.3389/fcell.2022.875468

**Published:** 2022-12-08

**Authors:** Carla Liaci, Mattia Camera, Valentina Zamboni, Gabriella Sarò, Alessandra Ammoni, Elena Parmigiani, Luisa Ponzoni, Enis Hidisoglu, Giuseppe Chiantia, Andrea Marcantoni, Maurizio Giustetto, Giulia Tomagra, Valentina Carabelli, Federico Torelli, Mariaelvina Sala, Yuchio Yanagawa, Kunihiko Obata, Emilio Hirsch, Giorgio R. Merlo

**Affiliations:** ^1^ Department of Molecular Biotechnologies and Health Sciences, University of Turin, Turin, Italy; ^2^ Neuroscience Institute Cavalieri Ottolenghi, Orbassano, Italy; ^3^ Neuroscience Institute, Consiglio Nazionale Ricerche, Milan, Italy; ^4^ Department of Drug Science, NIS Center, University of Turin, Turin, Italy; ^5^ Department of Neuroscience and National Institute of Neuroscience, University of Turin, Turin, Italy; ^6^ Institute for Physiology I, Medical Faculty, Albert-Ludwigs-University Freiburg, Freiburg, Germany; ^7^ Faculty of Biology, Albert-Ludwigs-University Freiburg, Freiburg, Germany; ^8^ Department of Genetic Behavioral Neuroscience, Gunma University, Maebashi, Japan; ^9^ RIKEN Brain Science Institute, Wako, Saitama, Japan

**Keywords:** inhibitory neurons, neuronal migration, Rho GTPases, ARHGAP15, RAC1, epilepsy

## Abstract

GTPases of the Rho family are components of signaling pathways linking extracellular signals to the control of cytoskeleton dynamics. Among these, RAC1 plays key roles during brain development, ranging from neuronal migration to neuritogenesis, synaptogenesis, and plasticity. RAC1 activity is positively and negatively controlled by guanine nucleotide exchange factors (GEFs), guanosine nucleotide dissociation inhibitors (GDIs), and GTPase-activating proteins (GAPs), but the specific role of each regulator *in vivo* is poorly known. ARHGAP15 is a RAC1-specific GAP expressed during development in a fraction of migrating cortical interneurons (CINs) and in the majority of adult CINs. During development, loss of ARHGAP15 causes altered directionality of the leading process of tangentially migrating CINs, along with altered morphology *in vitro*. Likewise, time-lapse imaging of embryonic CINs revealed a poorly coordinated directional control during radial migration, possibly due to a hyper-exploratory behavior. In the adult cortex, the observed defects lead to subtle alteration in the distribution of CALB2-, SST-, and VIP-positive interneurons. Adult *Arhgap15-*knock-out mice also show reduced CINs intrinsic excitability, spontaneous subclinical seizures, and increased susceptibility to the pro-epileptic drug pilocarpine. These results indicate that ARHGAP15 imposes a fine negative regulation on RAC1 that is required for morphological maturation and directional control during CIN migration, with consequences on their laminar distribution and inhibitory function.

## Introduction

Neuronal networks within the adult cerebral cortex are progressively established during development and postnatal life *via* extensive neuronal migration, neuritogenesis, and synaptogenesis in a highly temporally and spatially coordinated fashion. These cellular processes share common cellular mechanisms regulating the continuous and dynamic reorganization of the growth cone, a highly polarized structure at the tip of the leading process, which is essential to sense guidance cues, establish a trajectory, and orient the cell body (Murakoshi et al., 2011; Vitriol and Zheng, 2012). These dynamic processes are driven by extensive polymerization/depolymerization, branching, and severing of the actin filaments (Nadarajah et al., 2001; Cooper, 2008, 2013). The Rho family of small GTPases controls the spatial and dynamic changes in neuronal actin cytoskeleton organization (Tcherkezian and Lamarche-Vane, 2007; Heasman and Ridley, 2008; Govek et al., 2011; Gonzalez-Billault et al., 2012; Marín, 2013). Rho GTPases regulate protrusion, retraction, and adhesion at the growth cone of immature neurons through the control of microtubule stability and actin filament polymerization/depolymerization, actomyosin contractility, and engagement of intracellular adhesion and anchoring mechanisms (Gomez & Letourneau, 2014). Rho GTPases link extracellular cues to motility responses, as shown, for instance, in migrating cerebellar granule cells, in which the conditional inactivation of RAC1 phenocopies the defects observed in *Sema6A* and *Plxna2* knock-out (KO) mice (Tahirovic et al., 2010; Renaud and Chédotal, 2014). Broadly speaking, the activity of RAC1/RAC3 and CDC42 is associated with attractive growth cone turning, whereas the activity of RHOA is associated with responsiveness to repulsive cues (Luo, 2000). RAC1 is expressed in the embryonic and adult brain; embryonic RAC1 is mainly localized at the growth cone of migrating neurons (Hall and Lalli, 2010; Tahirovic et al., 2010) and is essential for neuronal migration (Chen et al., 2007; Govek et al., 2011; Heasman and Ridley, 2008; Kawauchi, 2015; Yang et al., 2012) and maturation (Gomez and Letourneau, 2014), both *in vitro* and *in vivo*. In mice, the *Rac1* whole-body KO is embryonically lethal (Chen et al., 2007, 2009), while the *Syn1-cre*-mediated conditional deletion of *Rac1* (named *Rac-N*) in neurons leads to migration, differentiation, and connectivity defects (Corbetta et al., 2008, 2009; Pennucci et al., 2011; Vaghi et al., 2014). Mice with both *Rac-N* and *Rac3* null mutations show defective migration and maturation of cortical and hippocampal inhibitory neurons, severe neurological and cognitive deficits, and spontaneous epilepsy, while the single disruption of *Rac3* does not cause evident defects (Corbetta et al., 2008, 2009; Pennucci et al., 2011; Vaghi et al., 2014). GTPases cycle between an inactive GDP-bound and an active GTP-bound state, a binary switch that is tightly regulated by multiple guanine nucleotide exchange factors (GEFs), GTPase-activating proteins (GAPs), and guanine nucleotide dissociation inhibitors (GDIs) (Peck et al., 2002; Watabe-Uchida et al., 2006). The GAP protein ARHGAP15 is a RAC1-specific negative regulator whose overexpression results in an increased actin stress fibers formation and cell contraction (Seoh et al., 2003; Costa et al., 2011). The GAP domain of ARHGAP15 binds the C-terminal half of RAC1 in a nucleotide-independent manner, promoting the RAC1 GDP-bound state and the consequent switch-off of the downstream pathway. A novel biochemical mechanism of RAC1 inactivation by ARHGAP15 may involve the interaction and mutual antagonism with PAK1, a well-known RAC1 effector (Radu et al., 2013). Animal models revealed specific functions of RAC1 GTPase in the development of inhibitory networks (Corbetta et al., 2008, 2009; Pennucci et al., 2011; Vaghi et al., 2014)*.* The principal inhibitory neurons in the cortex are the GABAergic cortical interneurons (CINs) derived from progenitors that reside in the ventral telencephalon of the embryonic brain, namely the ganglionic eminences, and in the preoptic area (POA) (Gelman et al., 2012). The ganglionic eminences appear around E11 and can be subdivided into the medial ganglionic eminence (MGE), the caudal ganglionic eminence (CGE), and the lateral ganglionic eminence (LGE) (DeDiego et al., 1994; de Carlos et al., 1996; Anderson et al., 1997; Brunstrom et al., 1997; Tamamaki et al., 1997). CINs that colonize the cortex derive mainly from NKX2.1-positive progenitors in the MGE and POA, while the LGE mostly contributes to striatal and olfactory bulb interneurons*.* After exiting the cell cycle, immature CINs begin to migrate tangentially to reach the cortex and the hippocampus primordia following stereotyped routes (Tanaka et al., 2011; Kelsom and Lu, 2013). During this tangential migration, Rho GTPases and their regulatory network have been shown to link extracellular signals to directional control (de Curtis, 2014; Tivodar et al., 2015). Mice lacking RAC1 in MGE-derived cells exhibit a 50% reduction in the number of GABAergic CINs in the postnatal cortex (Vidaki et al., 2012). Once in the appropriate cortical areas, CINs reorient their trajectory by approximately 90°, leaving the tangential path and proceeding with radial migration to reach their final position (Guo and Anton, 2014; Hatanaka et al., 2016). Factors that control the migration of CINs into the cortical plate are poorly known, as only a few of them have been identified (*e.g*., neuregulins, sonic hedgehog, and the thalamocortical projections) (Flames et al., 2004; Baudoin et al., 2012; Alfonso et al., 2015; Zechel et al., 2016; Bartolini et al., 2017). Here, we provide evidence for the requirement of the RAC1-specific negative regulator ARHGAP15 in the control of CIN migration, morphology, and functionality. Specifically, *Arhgap15*-KO CINs show a disoriented leading process during both tangential and radial migration and do not undergo proper cortical lamination. Moreover, *Arhgap15*-KO mice show increased susceptibility to sporadic spontaneous and induced seizures, probably due to reduced CINs intrinsic excitability.

## Materials and methods

### Mouse strains

All animal procedures were approved by the local Animal Ethics Committee and the Ministry of Health. Animals were maintained according to institutional animal welfare guidelines and legislation, under veterinarian surveillance. The *Arhgap15* ^*LacZ/LacZ*^ mouse strain has been previously described (Costa et al., 2011; Zamboni et al., 2016). Heterozygous and homozygous mutant mice are born at normal Mendelian frequency, appear overall normal, are viable and fertile, mate at regular rates, and do not show evident neurological or motor impairments. Animals were maintained in a mixed c57/bl6 genetic background. The *GAD67-eGFP* reporter mouse strain was generated by homologous recombination, introducing the *enhanced-GFP* (eGFP) cDNA cassette into the murine *GAD1* locus, coding for GAD67 (glutamic acid-decarboxylase-67), which is expressed by GABAergic neurons starting from early developmental stages (DeDiego et al., 1994; Sakai and Miyazaki, 1997; Tamamaki et al., 2003). Heterozygous progeny was obtained from chimeric males and backcrossed to the C57BL/6 background. The loxP-flanked PGK-neo cassette, used as a selection marker for screening the recombinant embryonic stem cells, was successfully excised by mating *GAD67-eGFP* mice with *CAG-cre* transgenic mice (Tamamaki et al., 2003).

### Brain preparation for histological analysis

For the collection of postnatal brains, mice were anesthetized with Avertin (30 μl of pure Avertin in 400 μl of PBS) and transcardially perfused with 10 ml of PBS (pH 7.4) and 10 ml of 4% (w/v) PFA in PBS (pH 7.4, adjusted with NaOH). Brains were removed, post-fixed overnight at 4°C in 4% PFA, placed overnight at 4°C in 30% (w/v) sucrose in PBS for cryoprotection, embedded in OCT blocks, and stored at −80°C until analysis. OCT blocks were cut into 30 μm-thick coronal sections using a cryotome (Leica CM 1950). Free-floating sections were collected in PBS in multiwell plates and stored at −20°C in a cryoprotectant solution (30% (v/v) glycerol and 30% (v/v) ethylene glycol in 0.2 M phosphate buffer, pH 7.4) until processed. The range of sections used for analysis corresponds to coronal sections 44–54 of the reference Allen Brain Atlas (Allen Reference Atlas–Mouse Brain [brain atlas]. Available from atlas.brain-map.org). For the analysis, we selected neurons in the somatosensory cortical region. For the collection of embryonic brains, embryos were obtained through cesarean section at E11.5, 12.5, E14.5, or E17.5 (considering the day of the vaginal plug as E0.5) from anesthetized pregnant dams and transferred in PBS. Embryonic brains used for immunohistochemistry were dissected and fixed overnight at 4°C in 4% PFA, then placed overnight at 4°C in 30% (w/v) sucrose in PBS for cryoprotection, embedded in OCT blocks, and stored at -80°C until analysis. OCT blocks were cut into 15 μm-thick coronal sections and collected on super-adhesive glass slides.

### Primary cultures of cortical neurons

Round glass slides were incubated with 80% nitric acid overnight, washed with deionized water several times, sterilized by autoclaving, coated with 1 mg/ml poly-l-lysine (Sigma) in borate buffer (pH 8.5) in a 12-well plate, and washed again with deionized water. One day before establishing the culture, glass slides were rinsed in a MEM (Gibco) solution with 1% (v/v) pyruvate 100X (Gibco), 7% (w/v) glucose, 1% (v/v) Penicillin-Streptomycin, and 10% (v/v) horse serum (Gibco). *GAD67-eGFP* and *Arhgap15* ^*LacZ/LacZ*^ *;GAD67-eGFP* E15.5 embryos were used to establish primary cultures of cortical neurons. Embryonic heads were dissected in sterile conditions in a cold solution of 1% (v/v) HEPES in HBSS with calcium and magnesium (Gibco). Cortices were dissected free of the rest of the brain, deprived of the meninges, washed with a cold solution containing 1% Penicillin-Streptomycin and 1% HEPES in HBSS with calcium and magnesium (Gibco), and incubated in 500 μl of Trypsine 0.05% (Gibco). Cortices were washed 5 times in HBSS at room temperature and disaggregated in a solution containing DNAase (used 1:1,000; Promega) by pipetting. Cells were counted and 80.000 cells were plated on each glass slide in a 12-well plate containing Neurobasal medium (Gibco) additioned with 1% Penicillin-Streptomycin, 2% (v/v) B27 (Gibco), and 0.25% (v/v) GlutaMAX (Gibco). Neurons were incubated at 37°C in a 5% CO_2_ saturation atmosphere.

### Immunostaining and image analysis

Brain sections were washed three times in PBS, incubated for 1 h at room temperature with a blocking solution (10% goat or donkey serum and 0.2% Triton-X 100 in PBS), and incubated overnight at 4°C with the primary antibodies diluted in a solution composed of 0.5% Triton-X100 and 5% goat or donkey serum. Then, sections were incubated for 2 h at room temperature with fluorophore-conjugated secondary antibodies diluted in a solution composed of 0.2% Triton-X100 and 3% goat or donkey serum and washed three times in PBS. Finally, sections were counterstained with DAPI and mounted with Mowiol onto super-adhesive glass slides. Double-immunostainings performed using two antibodies from the same host species were performed using the Tyramide SuperBoost™ kit with AlexaFluor™ Tyramides (Invitrogen). Primary cortical cultures were fixed at 3, 10, and 18 days *in vitro* (DIV) with 4% PFA in PBS for 20 min at room temperature. Neurons were incubated for 1 h at room temperature with a blocking solution containing 5% goat serum and 0.1% Triton-X in PBS. The primary antibody was diluted in a solution containing 3% goat serum and 0.1% Triton-X in PBS and incubated overnight at 4°C. Secondary antibodies were incubated for 1 h at room temperature. Coverslips were mounted with Mowiol onto glass slides. Primary antibodies: rabbit anti-CALB2 (Calretinin; used 1:500; Swant, 7,697), rabbit anti-PVALB (Parvalbumin; used 1:1,000; Swant, PV27), goat anti-SST (Somatostatin; used 1:500; SantaCruz, sc-7819s), rabbit anti-β-GAL (β-Galactosidase; used 1:1,000; MP Biomedicals, SKU:085597-CF), and rabbit anti-VIP (Vasoactive intestinal peptide; used 1:500; Invitrogen, PA5-78224). Secondary antibodies: Alexa Fluor 488 donkey anti-rabbit IgG and AlexaFluor 568 goat anti-mouse IgG (used 1:500; Invitrogen). Slides were examined with a Leica SP8 confocal microscope. Raw images were digitally processed to normalize the background and optimize the contrast, rotated, and sized with ImageJ (NIH, Bethesda, Maryland; http://imagej.nih.gov/ij/). For expression and layering analysis, cortices were divided into 10 horizontal bins of equal thickness (bin one is the outermost and closest to the pia, bin 10 is the innermost and closest to the ventricle). For tangential migration analysis, polar plots were generated by using matplotlib *Python* library (https://matplotlib.org/stable/#). Morphological analysis on primary cultures was performed using ImageJ (NIH, Bethesda, Maryland; http://imagej.nih.gov/ij/); arborization of each neuron was quantified by performing Sholl analysis (Sholl, 1953) by ImageJ plugin Sholl Analysis Plugin (v1.0), Ghosh Lab Software (http://ghoshlab.org/software/index.html).

### Live imaging of radially migrating neurons in organotypic slice cultures

300 μm-thick brain slices were prepared by vibratome sectioning from E17.5 *GAD67-eGFP* and *Arhgap15* ^*LacZ/LacZ*^ *;GAD67-eGFP* embryos. Slices were kept in cold PBS for 20 min, then transferred in Neurobasal medium (Gibco) additioned with 1% Penicillin-Streptomycin, 2% B27 (Gibco), and 0.25% GlutaMAX (Gibco) and maintained in culture for 6 h at 37°C and 5% CO_2_. The organotypic slice cultures were imaged, while kept at 37°C and 5% CO_2_, by time-lapse video imaging for about 5 h with a frame interval of 5 min using a ×20 objective. The acquired movies were used to determine the migration trajectory of individual CINs. Videos were analyzed using the Manual Tracking plugin of ImageJ (NIH, Bethesda, Maryland; http://imagej.nih.gov/ij/) and Photoshop (Adobe). A virtual grid with lines perpendicular to the SVZ and the pial surfaces was superimposed in each frame. Neurons were considered to be in radial orientation if their leading process (the longest and widest forward branch) formed an angle between 0° and 25° with the perpendicular lines of the grid, as previously done (Martini et al., 2009). The paths were measured by tracking the movements of the leading process of each neuron throughout the entire time-lapse.

### EEG recording of awake animals

Mice were anesthetized with isoflurane (2% (v/v) in 1 L/min O_2_). Four screw electrodes (Bilaney Consultants GMBH) were inserted bilaterally through the skull (anteroposterior +2.0–3.0 mm, mediolateral 2.0 mm from bregma). A grounding electrode was placed into the nasal bone. The five electrodes were connected to a pedestal and fixed with acrylic cement (Palavit), as previously described (Manfredi et al., 2009). EEGs were recorded from eighteen (ten wild-type and eight *Arhgap15* ^*LacZ/LacZ*^) freely moving awake animals in a Faraday chamber, using a Power-Lab digital acquisition system (AD Instruments) with a sampling rate of 100 Hz and a resolution of 0.2 Hz. The basal cerebral activity was recorded continuously for 6 h. EEG tracings were analyzed and scored for the presence of rhythmic 4–6 Hz sharp waves of rhythmic spindle-like events (Erbayat-Altay et al., 2008), spikes and solitary spikes followed by slow waves activity, and trains of spikes. Spikes were recognized as having a duration <200 ms with a baseline amplitude set to 4.5 times the standard deviation of the EEG signal (determined during interspike activity periods). Repetitive spiking activity (trains of spikes) was defined by the presence of at least five consecutive spikes in less than 5 s (Berretta et al., 2022). The classification “trains of spikes” was attributed to mice showing at least four events. Spike activity was quantified using LabChart 8 (AD Instruments). Segments with movement artifacts or electrical noise were excluded from statistical analysis.

### Chemical induction of epilepsy

Eight (five females and three males) P90 mice for each genotype were transferred to individual cages in a quiet room. Atropine (1 mg/kg; Sigma) was administered by intraperitoneal injection to limit the peripheral side effects of pilocarpine. After 30 min, pilocarpine hydrochloride (350 mg/kg, Sigma) was administered by intraperitoneal injection. Animals were monitored every 10 min for 90 min after pilocarpine administration. We used a seizure staging system adapted from the established rodent seizure Racine’s scale (Racine, 1972): stage 0, no abnormality; stage 1, exploring, sniffing, and grooming ceased, becoming motionless; stage 2, forelimb and/or tail extension, appearance of rigid posture; stage 3, myoclonic jerks of the head and neck, with brief twitching or repetitive movements with head bobbing; stage 4, forelimb clonus and partial or occasional rearing and falling; stage 5, forelimb clonus, continuous rearing and falling; stage 6, tonic-clonic movements with loss of posture tone; stage 7, death.

### Whole-cell patch-clamp recording

For acute slices, 4 *GAD67-eGFP* and 4 *Arhgap15* ^*LacZ/LacZ*^ *;GAD67-eGFP* P150 mice were killed by cervical dislocation. Brains were removed and placed at 4°C in oxygenated (95%O_2_–5%CO_2_) adapted artificial cerebrospinal fluid (ACSF), containing 120 mM choline chloride, 3.5 mM KCl, 0.5 mM CaCl_2_, 6 mM MgSO_4_, 1.25 mM NaH_2_PO_4_, 25 mM d-glucose and 25 mM NaHCO_3_. Somatosensory cortex coronal slices (300 μm) were cut in ice-cold ACSF using a vibratome (Microm HM 650 V, Thermo Scientific) and subsequently placed for 30 min in ACSF containing 120 mM NaCl, 3.5 mM KCl, 25 mM, NaHCO_3_, 25 mM d-glucose, 2.5 mM CaCl_2_, 1.3 mM MgSO_4_, and 1.25 mM NaH_2_PO_4_, at 32 °C. Slices were kept at room temperature for at least 1 h before recording. Patch electrodes of borosilicate glasses (Hilgenberg, Mansfield, Germany) were pulled to a final resistance of 5–9 MΩ. For current-clamp recordings in both brain slice and primary cultured neurons, the internal solution contained: 135 mM gluconic acid (potassium salt: K-gluconate), 5 mM NaCl, 2 mM MgCl_2_, 10 mM HEPES, 0.5 mM EGTA, 2 mM ATP-Tris, and 0.4 mM Tris-GTP.

Patch-clamp recordings from CINs (somatosensory cortex, layer IV-VI) were performed in whole-cell configuration using an EPC-10 amplifier (HEKA Elektronic, Lambrecht, Germany). Traces were sampled at 10 kHz and filtered using a low-pass Bessel filter set at 2 kHz. All the experiments were performed at room temperature (22–24°C). Resting membrane potential (Vrest) and membrane capacitance (Cm) were routinely acquired when the whole-cell patch-clamp configuration was established. The membrane time constant (tm) was calculated by Clampfit software following a step current injection of −30 pA. The membrane capacitance (Cm) was calculated by applying the formula Cm = τm/Rin.

The action potential (AP) parameters were obtained by analyzing a series of spikes recorded during tonic firing of 1–2 min duration. Tonic firing was elicited by depolarizing membranes with a minimum amount of current corresponding to the rheobase value (Marcantoni et al., 2014). After reaching steady-state condition during tonic firing, at least five APs were selected and averaged for each cell, then the measures of AP peak amplitude, half-width, maximum rising slope, and maximum repolarizing slope were performed with Clampfit software (Axon Instruments). The peak amplitude of AP was measured from the threshold to the AP peak and the half-width was calculated at half-maximal AP height.

To analyze the relationship between firing frequency and injected current, the membrane potential was adjusted to −70 mV and then 20 pulses of increasing intensity (from -30–160 pA, 500 ms duration) were injected. The mean firing frequency of each current step was calculated as the number of spikes per second. The rheobase was determined as the minimum amount of current required to trigger one spike. Input resistance (Rin) was calculated in a linear region of the membrane voltage-injected current curve centered at the holding potential (−70 mV), through the injection of hyperpolarizing and depolarizing current steps (from −30 to 30 pA; 10 pA steps).

For primary neuronal cultures, the extracellular solution for current recordings (Tyrode’s solution) contained: 2 mM CaCl_2_, 10 mM HEPES, 130 mM NaCl, 4 mM KCl, 2 mM MgCl_2_, and 10 mM d-glucose (Tomagra et al., 2019).

Patch-clamp recordings were performed using an EPC-9 amplifier (HEKA Elektronic, Lambrecht, Germany) and pClamp software (Molecular Devices, Silicon Valley, CA, United States). Analysis of firing activity was performed with Clampfit software (Axon Instruments).

### Statistical analysis

For the statistical comparisons, GraphPad Prism software (GraphPad Software Inc.) was used. For each experiment, the statistical test used is reported in the figure legends. Shapiro-Wilk and Kolmogorov-Smirnov tests were used to test for normality, F test was used to test for equality of variance, and results were evaluated to choose the appropriate statistical test. The results are shown as mean ± Standard Error of Mean (SEM). The threshold for statistical significance was set at *p* < 0.05.

## Results

### Expression of ARHGAP15 in embryonic and adult CINs

**FIGURE 1:**
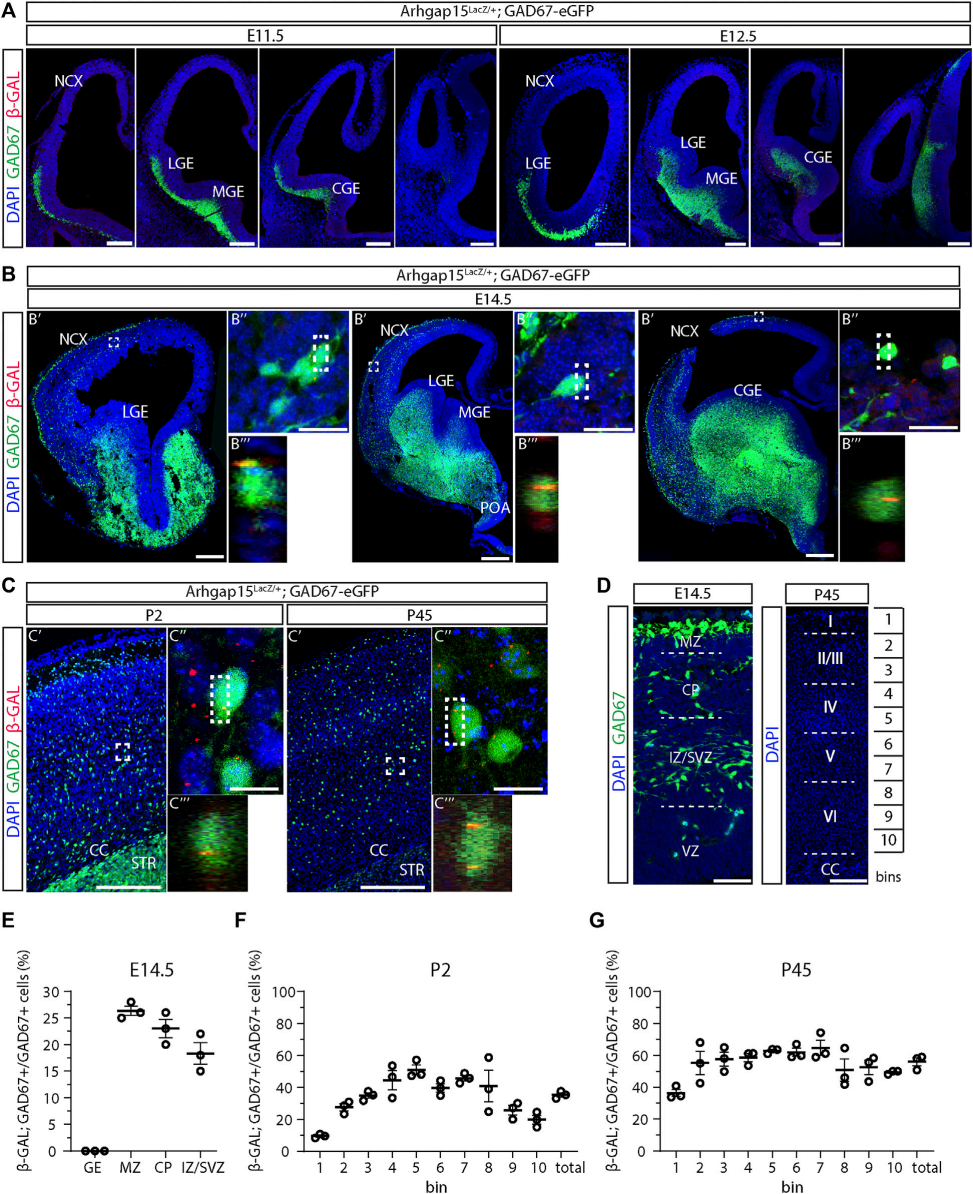
Expression of ARHGAP15 in embryonic and adult CINs. **(A)** Maximum intensity projections of z-stack images (5 serial image planes; z step size = 2 µm) of coronal sections of E11.5 and E12.5 *Arhgap15* ^*LacZ/+*^;*GAD67-eGFP* brains immunostained for *β*-GAL. The left-right order of the images recapitulates the rostral-caudal axis. Scale bars: 200 µm. **(B)** Coronal sections of E14.5 *Arhgap15* ^*LacZ/+*^;*GAD67-eGFP* brains. (B′) Confocal optical sections of E14.5 *Arhgap15* ^*LacZ/+*^;*GAD67-eGFP* brains immunostained for *β*-GAL; (B″) Maximum intensity projections of z-stack images (20 serial image planes; z step size = 0.5 µm) of the regions inside the dashed boxes in B′; (B‴) Orthogonal projections of the regions inside the dashed boxes in B″ showing the co-localization of the GAD67 and *β*-GAL signals. Scale bars: 200 µm in B′ and 20 µm in B″. **(C)** Coronal sections of P2 and P45 *Arhgap15* ^*LacZ/+*^;*GAD67-eGFP* brains; (C′) Maximum intensity projections of z-stack images (5 serial image planes; z step size = 2 µm) of the somatosensory cortex of P2 and P45 *Arhgap15* ^*LacZ/+*^;*GAD67-eGFP* brains immunostained for *β*-GAL; (C″) Maximum intensity projections of z-stack images (20 serial image planes; z step size = 0.5 µm) of the regions inside the dashed boxes in C′; (C‴) Orthogonal projections of the regions inside the dashed boxes in C″ showing the co-localization of the GAD67 and *β*-GAL signals. Scale bars: 200 µm in C′ and 20 µm in C″. **(D)** Representation of the different subregions of the E14.5 neocortex (left) and the correspondence between bins and cortical layers in the adult (P45) cortex (right). Scale bars: 50 µm (left) and 200 µm (right). **(E–G)** Percentage of *β*-GAL/GAD67 double-positive cells over the total of GAD67-positive cells across the E14.5 neocortex subregions **(E)**, early postanal (P2) **(F)**, and adult (P45) cortical bins **(G)**. *n* = 3 E14.5 embryos, 3 P2 and 3 P45 mice. At least 100 cells were evaluated for *β*-GAL expression in each subregion of each brain. Data are presented as mean ± SEM. NCX, neocortex; LGE, lateral ganglionic eminence; MGE, medial ganglionic eminence; CGE, caudal ganglionic eminence; POA, preoptic area; CC, corpus callosum; STR, striatum; MZ, marginal zone; CP, cortical plate; IZ, intermediate zone; SVZ, subventricular zone; VZ, ventricular zone; GE, ganglionic eminence.

**FIGURE 2:**
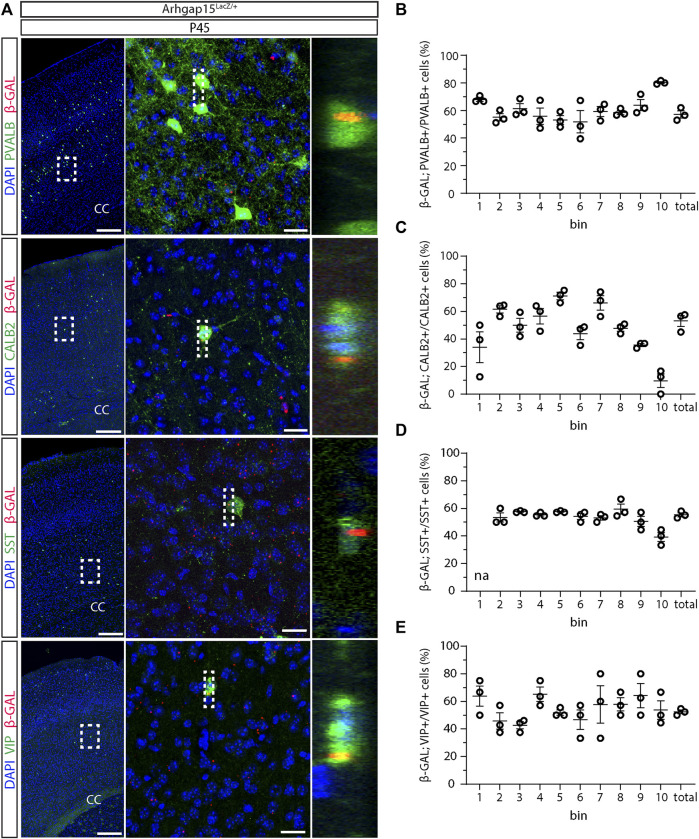
Expression of ARHGAP15 across the major CIN subtypes. **(A)** In the left column, maximum intensity projections of z-stack images (10 serial image planes; z step size = 2 µm) of the somatosensory cortex of P45 *Arhgap15* ^*LacZ/+*^;*GAD67-eGFP* brains immunostained for *β*-GAL and PVALB, CALB2, SST, or VIP. In the central column, maximum intensity projections of z-stack images (10 serial image planes; z step size = 0.5 µm) of the regions inside the dashed boxes on the left. In the right column, orthogonal projections of the regions inside the dashed boxes in the middle showing the co-localization of the PVALB, CALB2, SST, or VIP and the *β*-GAL signals. Scale bars: 200 µm (left), 20 µm (middle). **(B–E)** Percentage of *β*-GAL/PVALB **(B)**, *β*-GAL/CALB2 **(C)**, *β*-GAL/SST **(D)**, and *β*-GAL/VIP **(E)** double-positive cells over the total of PVALB, CALB2, SST, and VIP-positive cells, respectively. *n* = 3 mice; about 120 cells were evaluated for *β*-GAL expression for each bin in each brain. Data are presented as mean ± SEM.

We previously reported ARHGAP15 expression in the cortex, hippocampus, and olfactory bulbs of early postnatal and adult mice (Zamboni et al., 2016, 2018). To evaluate the expression of ARHGAP15 in embryonic and adult CINs, we examined the expression of the *LacZ* knock-in reporter in brain sections of *Arhgap15* ^*LacZ/+*^ *;GAD67-eGFP* animals by immunostaining for β-GAL. *LacZ* expression was not detected in E11.5 and E12.5 brains (Figure 1A). In sections of E14.5 brains, *LacZ* expression was detected in 26 ± 0.9%, 23 ± 1.7%, and 18 ± 2% of CINs in the marginal zone (MZ), cortical plate (CP), and intermediate zone (IZ)/subventricular zone (SVZ), respectively (Figures 1B,E). No *LacZ* expression was detected in the ganglionic eminences, indicating that embryonic CINs start expressing ARHGAP15 only after they start migrating and enter the neocortex (NCX). In sections of early postanal (P2) and adult (P45) brains, *LacZ* expression was detected in 35 ± 1.3% and 56 ± 2.6% of CINs, respectively (Figures 1C,F,G). To determine the expression of ARHGAP15 in the major CIN subtypes, we carried out double immunostainings for β−GAL and either PVALB, CALB2, SST, or VIP on sections of P45 *Arhgap15* ^*LacZ/+*^ brains. The results show that ARHGAP15 is expressed by 57 ± 3%, 53 ± 4%, 53 ± 1%, and 52 ± 1% of PVALB-, CALB2-, SST-, and VIP-positive neurons, respectively (Figures 2A–E). These results indicate that ARHGAP15 starts to be expressed in migrating CINs between the embryonic stages E13.5 and E14.5, and it is expressed by most (about 60%) of adult CINs, not being specific for any of the major CIN subtypes.

### Altered morphology of *Arhgap15* ^*LacZ/LacZ*^ CINs *in vitro*

**FIGURE 3:**
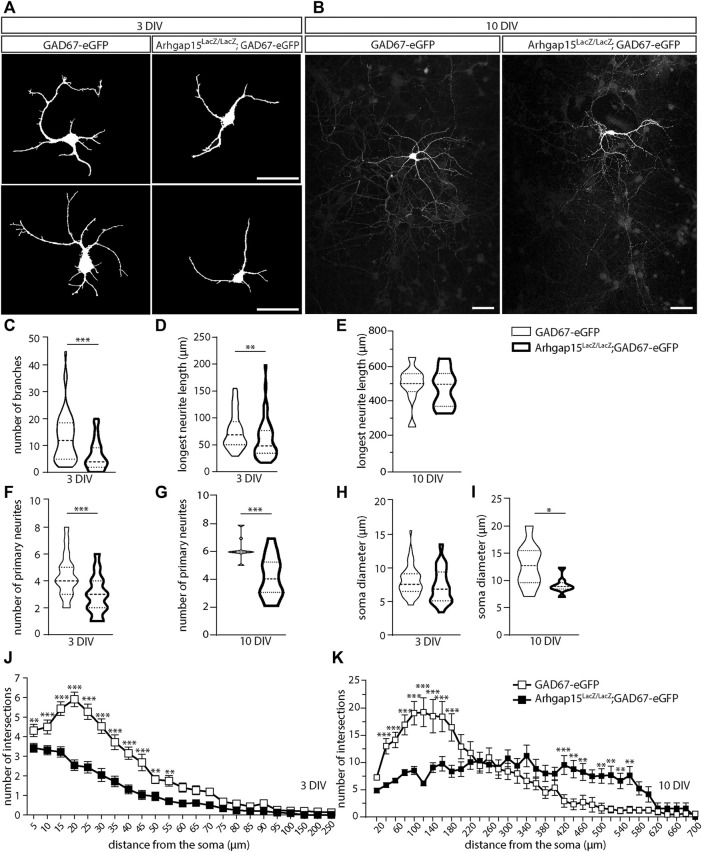
Morphological analysis of primary CINs. **(A,B)** Representative fluorescence micrographs of eGFP-positive primary CINs from *GAD67-eGFP* (control) and *Arhgap15* ^*LacZ/LacZ*^;*GAD67-eGFP* mice after 3 **(A)** and 10 **(B)** DIV. Scale bars: 20 µm. **(C)** Number of branches in *GAD67-eGFP* and *Arhgap15* ^*LacZ/LacZ*^;*GAD67-eGFP* primary CINs after 3 DIV. p = 8 × 10^−6^. **(D,E)** Length of the longest neurite in *GAD67-eGFP* and *Arhgap15* ^*LacZ/LacZ*^;*GAD67-eGFP* primary CINs after 3 (D; p = 7 × 10^−3^) and 10 (E; *p* = 0.75) DIV. **(F,G)** Number of primary neurites in *GAD67-eGFP* and *Arhgap15* ^*LacZ/LacZ*^;*GAD67-eGFP* primary CINs after 3 (F; *p* = 3 × 10^−6^) and 10 (G; *p* = 4 × 10^−4^) DIV. **(H,I)** Average diameter of the soma of *GAD67-eGFP* and *Arhgap15* ^*LacZ/LacZ*^;*GAD67-eGFP* primary CINs after 3 (H; *p* = 0.08) and 10 (I; *p* = 0.04) DIV. **(J,K)** Sholl analysis showing the overall complexity of arborization in *GAD67-eGFP* and *Arhgap15* ^*LacZ/LacZ*^;*GAD67-eGFP* primary CINs after 3 DIV (J; p (from 5 to 250 µm) = 0.03, 6 × 10^−3^, 2 × 10^−6^, 1 × 10^−6^, 1 × 10^−6^, 3 × 10^−6^, 3 × 10^−5^, 5 × 10^−6^, 6 × 10^−4^, 5 × 10^−3^, 2 × 10^−3^, 7 × 10^−3^, 0.02, 7 × 10^−3^, 0.05, 0.04, 0.06, 0.01, 0.17, 0.30, 0.03, 0.10, 0.10) and 10 DIV (K; p (from 10 to 700 µm) = 0.23, 2 × 10^−4^, 2 × 10^−4^, 1 × 10^−5^, 1 × 10^−6^, 2 × 10^−6^, 1 × 10^−5^, 5 × 10^−5^, 0.04, 0.39, 0.70, 0.51, 0.88, 0.23, 0.49, 0.20, 0.18, 0.07, 0.20, 9 × 10^−4^, 0.20, 1 × 10^−3^, 5 × 10^−3^, 8 × 10^−3^, 3 × 10^−3^, 2 × 10^−3^, 6 × 10^−3^, 1 × 10^−3^, 0.04, 0.14, 0.80, 0.59, 0.59, 0.64, 0.79). At least 70 (3 DIV) and 35 (10 DIV) neurons from 3 independent primary cultures were analyzed for each genotype. Data are presented as mean ± SEM. *p*-values were calculated using unpaired Mann–Whitney test **(C–I)** and unpaired multiple *t*-test corrected for False Discovery Rate (<1%) **(J,K)**. * = *p*< 0.05, ** = *p*< 0.01, *** = *p*< 0.001. DIV, days *in vitro*.

RAC1 is critical for neuritogenesis during development (Sayyad et al., 2016). To determine whether loss of ARHGAP15, hence hyperactive RAC1, affects CINs morphology (*i.e.*, neurite elongation and complexity), we examined cultures of dissociated neurons from cortices of E15.5 *GAD67-eGFP* and *Arhgap15* ^LacZ/LacZ^;*GAD67-eGFP* embryos at 3 and 10 DIV. We selected eGFP-positive neurons and examined their neurites length and complexity (Figures 3A,B). At 3 DIV, *Arhgap15* ^*LacZ/LacZ*^ eGFP-positive neurons displayed a reduced number of branches (Figure 3C). At 3 DIV, but not at 10 DIV, *Arhgap15* ^*LacZ/LacZ*^ eGFP-positive neurons also showed a reduced length of the longest neurite compared to the *GAD67-eGFP* (Figures 3D,E). At both 3 and 10 DIV, *Arhgap15* ^*LacZ/LacZ*^ eGFP-positive neurons showed a reduced number of primary neurites (Figures 3F,G). Moreover, at 10 DIV, but not at 3 DIV, a reduction in soma diameter was also observed in *Arhgap15* ^*LacZ/LacZ*^ eGFP-positive neurons (Figures 3H,I). Sholl analysis revealed that, at 3 DIV, *Arhgap15* ^*LacZ/LacZ*^ eGFP-positive neurons showed a lower number of intersections at various distances from the cell body (Figure 3J), indicating a reduced efficiency in neuritogenesis and branching, whereas at 10 DIV they showed a lower number of intersections in the region more proximal to the soma, but a higher number of distal intersections (Figure 3K). These results suggest that *Arhgap15* ^*LacZ/LacZ*^ CINs fail to undergo an efficient neuritogenesis and branching in the first maturation steps *in vitro*, but they later develop an increased number and/or branching of secondary neurites, possibly to compensate for their defective number of primary neurites.

To confirm that cultured eGFP-positive neurons express ARHGAP15, we performed immunostainings for β−GAL on primary cultures derived from *Arhgap15* ^*LacZ/+*^;*GAD67-eGFP* embryos at 3, 10, and 18 DIV. This analysis showed that about half of eGFP-positive neurons express ARHGAP15 also in culture (Supplementary Figure S1).

### Defective orientation of tangentially migrating *Arhgap15* ^*LacZ/LacZ*^ CINs

**FIGURE 4:**
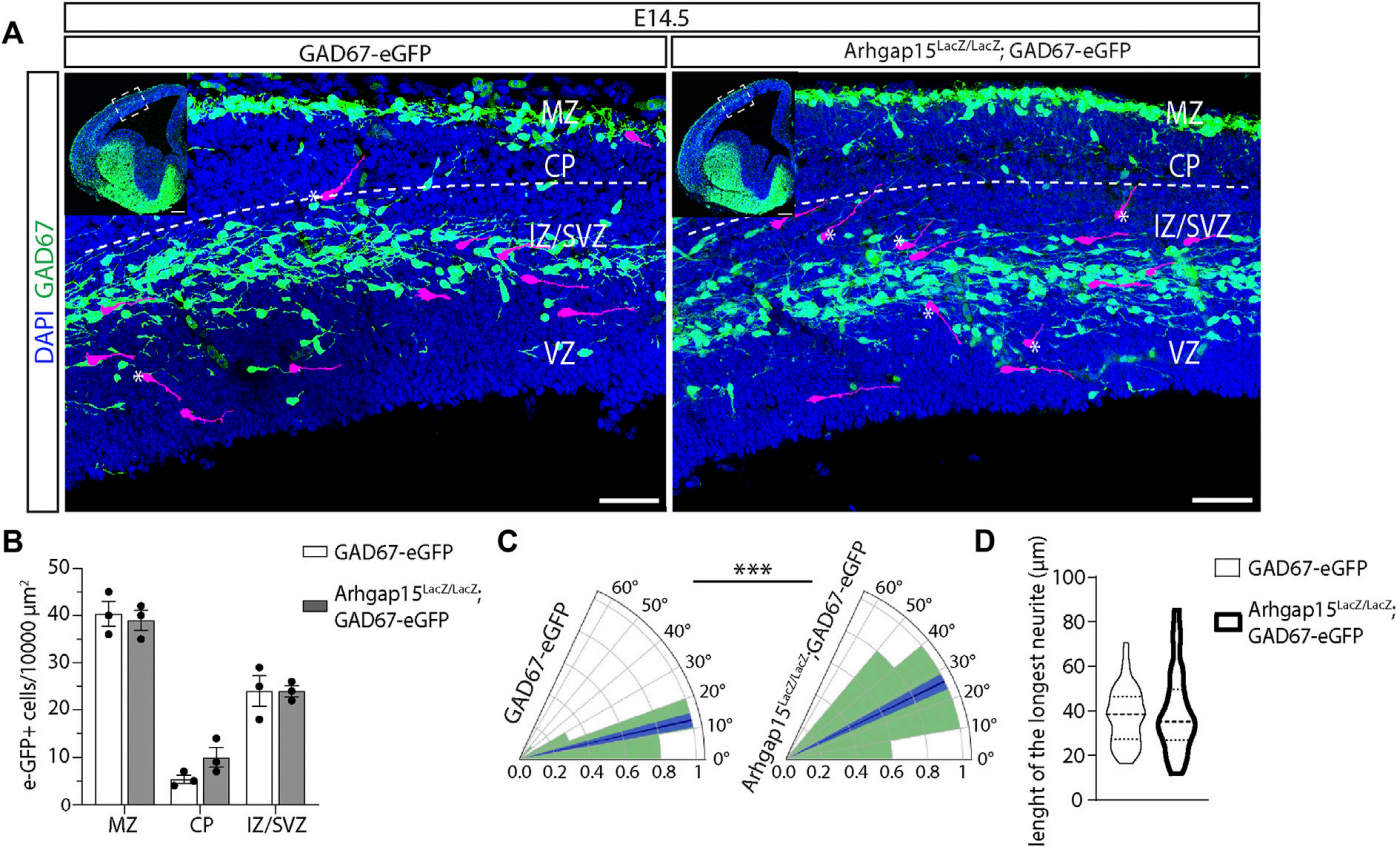
CIN tangential migration in the embryonic cortex. **(A)** Maximum intensity projections of z-stack images (10 serial image planes; z step size = 1 µm) of the neocortex of E14.5 *GAD67-eGFP* (control) and *Arhgap15* ^*LacZ/LacZ*^;*GAD67-eGFP* brains. Inserts show the analyzed cortical region. Some eGFP-positive neurons are highlighted in magenta to improve visualization. The dashed line represents the canonical CINs tangential migratory route, parallel to the pia. * indicates tangentially migrating CINs deviating from the canonical trajectory. Scale bars: 50 µm (main panels) and 200 µm (inserts). **(B)** Average density of eGFP-positive neurons in the MZ, CP, and IZ/SVZ of E14.5 *GAD67-eGFP* and *Arhgap15* ^*LacZ/LacZ*^;*GAD67-eGFP* embryos. p (from MZ to IZ/SVZ) = 0.8, 0.2, 0.99. **(C)** Polar plots showing the distribution (expressed as normalized frequency) of angles measured between tangentially migrating CIN leading processes and the line parallel to the pia in E14.5 *GAD67-eGFP* and *Arhgap15* ^*LacZ/LacZ*^;*GAD67-eGFP* brains. The dark blue line represents the average angle, the light blue area represents SEM. *p* = 2.9 × 10^−4^. **(D)** Average length of the longest neurite in E14.5 *GAD67-eGFP* and *Arhgap15* ^*LacZ/LacZ*^;*GAD67-eGFP* migrating CINs. p = 0.16. At least 50 neurons from 3 different embryos were analyzed for each genotype. *p*-values were calculated using unpaired Mann–Whitney test. *** = *p*< 0.001. MZ, marginal zone; CP, cortical plate; IZ, intermediate zone; SVZ, subventricular zone; VZ, ventricular zone.

Neuronal migration requires extensive cytoskeletal reorganization at the growth cone, a process in which small Rho GTPases play a critical role (Liaci et al., 2021). Since interneurons cover a longer and more complex migratory path as compared to other neurons (Cooper, 2013) and were shown to express ARHGAP15 during their migration from the GE to the NCX (Figure 1B), we looked at their cortical migration. We determined the number and position of tangentially migrating CINs in the cortices of *GAD67-eGFP* and *Arhgap15* ^*LacZ/LacZ*^;*GAD67*-*eGFP* embryos by analyzing coronal sections of E14.5 brains (Figure 4A). No significant changes in the number of tangentially migrating CINs were detected between the two genotypes (Figure 4B). We examined the orientation of eGFP-positive neurons by determining the angle between their leading process and the canonical direction of their tangential migration (*i.e.*, parallel to the pial and ventricular surfaces). In the *Arhgap15* ^*LacZ/LacZ*^ *;GAD67-eGFP* embryonic cortex, the leading process of migrating CINs displayed a mean angle relative to the tangential trajectory significantly wider than that of *GAD67-eGFP* control neurons (Figure 4C), indicating that *Arhgap15* ^*LacZ/LacZ*^ CINs tend to deviate from the canonical tangential direction of migration. No differences were observed comparing the length of the leading processes of *GAD67-eGFP* and *Arhgap15* ^*LacZ/LacZ*^;*GAD67*-*eGFP* tangentially migrating CINs (Figure 4D). Thus, the absence of ARHGAP15 alters the control of leading process directionality during tangential migration.

### Defective orientation of radially migrating *Arhgap15* ^*LacZ/LacZ*^ CINs

**FIGURE 5:**
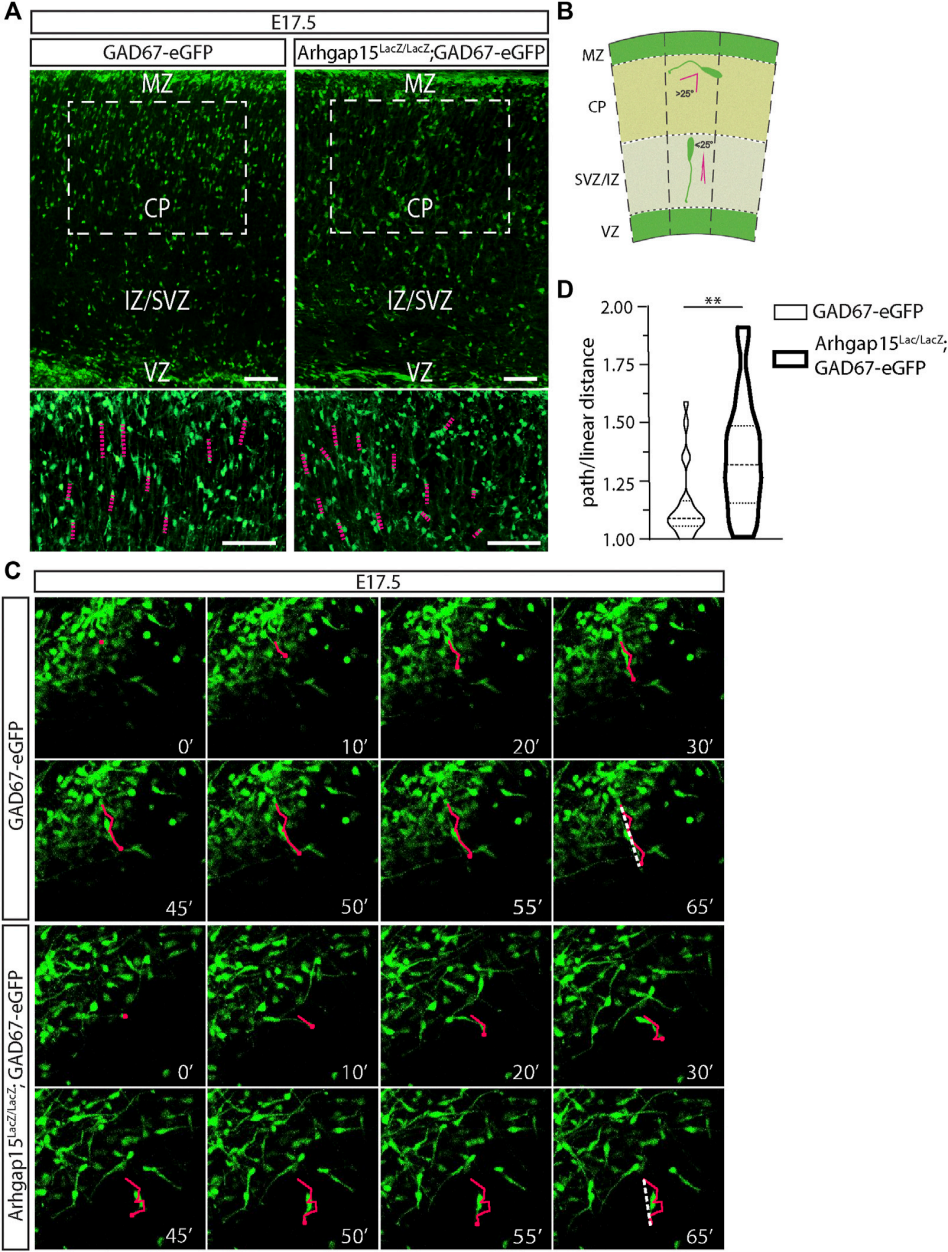
CIN radial migration in the embryonic cortex. **(A)** Maximum intensity projections of z-stack images (5 serial image planes; z step size = 1 μm) of E17.5 *GAD67-eGFP and Arhgap15* ^*LacZ/LacZ*^;*GAD67-eGFP* mouse cortices. Scale bars: 100 µm. The bottom panels show a higher magnification of the region in the dashed box. Scale bars: 100 µm. **(B)** Schematic representation of the criteria used to discriminate between tangential and radial migrating CINs. The dashed lines indicate perpendicular lines to both the pia and ventricle. Leading processes forming an angle higher than 25° with the dashed line are classified as tangentially migrating CINs, while the ones forming an angle lower than (or equal to) 25° are classified as radially migrating CINs. **(C)** Frames of representative time-lapse videos (Suppl. Videos 1 and 2) of E17.5 *GAD67-eGFP and Arhgap15* ^*LacZ/LacZ*^;*GAD67-eGFP* mouse cortices at different time points (from 0 to 65 min). In each frame, the tip of the leading edge of representative neurons was marked to reconstruct the migratory path. In the last frame (65′), the dashed line shows the linear distance covered by the leading edge. **(D)** Average ratio between the path and the linear distance covered by *GAD67-eGFP and Arhgap15* ^*LacZ/LacZ*^;*GAD67-eGFP* radially migrating neurons in the time window of the time-lapse video. At least 50 neurons from 3 different mice were analyzed for each genotype. *p* = 0.007. Data are presented as mean ± SEM. *p*-value was calculated using unpaired Mann–Whitney test. ** = *p*< 0.01. MZ, marginal zone; CP, cortical plate; IZ, intermediate zone; SVZ, subventricular zone, VZ, ventricular zone.

After E15.5, eGFP-positive tangentially migrating neurons present in the MZ and the IZ/SVZ routes have colonized all the cortical areas (Martini et al., 2009). Subsequently, they activate a different migration mode, namely the radial migration, perpendicular to the cortical and ventricular surfaces. Specifically, neurons in the MZ and IZ/SVZ migrate deeply to occupy the CP. Only a small fraction of CINs migrate through the subplate (Tanaka and Nakajima, 2012; Marín, 2013). We looked at CINs trajectory during their radial migration in coronal sections of E17.5 *GAD67-eGFP* and *Arhgap15* ^*LacZ/LacZ*^ *;GAD67-eGFP* brains. The magnifications of CPs suggested that radially migrating CINs may have altered trajectories with respect to the canonical radial direction (Figure 5A). To precisely determine CINs trajectory in the cortical primordium, we monitored single eGFP-positive CINs by time-lapse video imaging in 300 μm-thick slices of E17.5 *GAD67-eGFP* and *Arhgap15* ^*LacZ/LacZ*^ *;GAD67-eGFP* cortices maintained in organotypic cultures. We examined the trajectory of *GAD67-eGFP* and *Arhgap15* ^*LacZ/LacZ*^ *;GAD67-eGFP* CINs after their tangential-to-radial switch and tracked their leading process movements for at least 100 min. Most of them were stationary after 65 min. The criteria used for identifying radially migrating neurons are graphically illustrated in Figure 5B (Martini et al., 2009). We calculated the ratio between the path length and linear distance between the initial and final positions of the leading process of each neuron (Figure 5C, Supplementary Videos S1, 2). We observed that *Arhgap15* ^*LacZ/LacZ*^ *;GAD67-eGFP* neurons deviate from the physiological radial direction of migration significantly more often than *GAD67-eGFP* (Figure 5D). This result suggests a loss of directional control (or a gain of directional flexibility) in *Arhgap15-*KO CINs, in accordance with data shown in Figure 4.

### Altered distribution of CALB2-, SST-, and VIP-positive neurons across the *Arhgap15* ^*LacZ*/LacZ^ adult cortical layers

**FIGURE 6:**
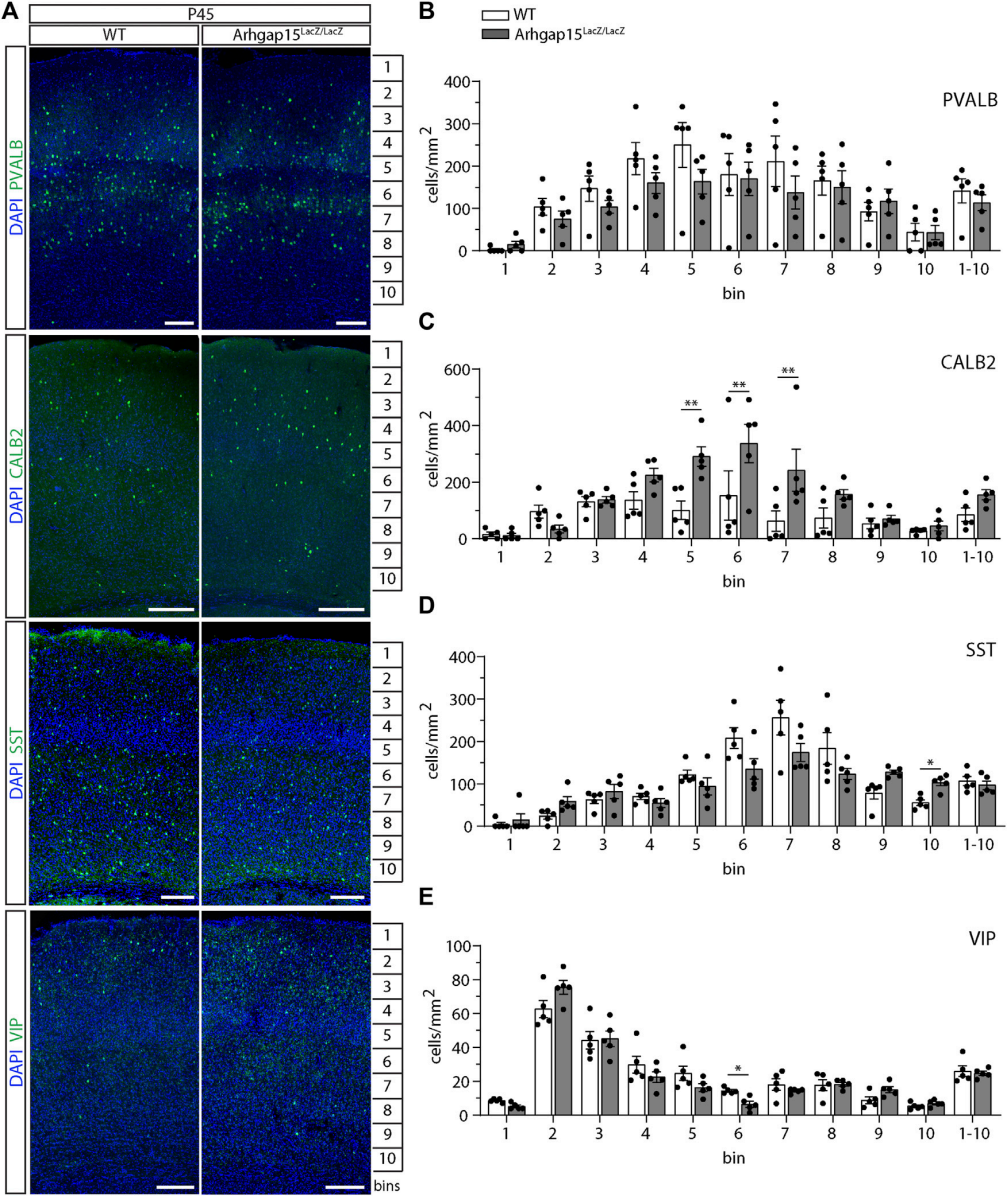
CIN distribution across layers in the adult cortex. **(A)** Maximum intensity projections of z-stack images (10 serial image planes; z step size = 2 μm) of coronal sections of WT and *Arhgap15* ^*LacZ/LacZ*^ P45 somatosensory cortices immunostained for PVALB, CALB2, SST, and VIP. **(B–E)** Average density of PVALB- **(B)**, CALB2- **(C)**, SST- **(D)**, and VIP- **(E)** positive neurons in each bin of P45 WT and *Arhgap15* ^*LacZ/LacZ*^ somatosensory cortices. At least 4 sections from 5 different mice were analyzed for each genotype. p (PVALB) > 0.99 for all bins; p(CALB2, from bin 1 to 1-10) = 0.99, 0.71, 0.99, 0.47, 0.003, 0.004, 0.005, 0.52, 0.99, 0.99, 0.65; p (SST, from bin 1 to 1-10) = 0.78, 0.32, 0.78, 0.78, 0.78, 0.44, 0.60, 0.72, 0.18, 0.02, 0.78; p(VIP, from bin 1 to 1-10) = 0.07, 0.51, 0.99, 0.76, 0.59, 0.03, 0.77, 0.99, 0.33, 0.72, 0.98. Data are presented as mean ± SEM. *p*-values were calculated using unpaired multiple *t*-test corrected for multiple comparisons using the Holm-Sidak method. * = *p*< 0.05; ** = *p*< 0.01.

Given the alterations in CIN migration described above, we examined the laminar distribution of CINs in WT and *Arhgap15* ^*LacZ/LacZ*^ adult (P45) cortices. We examined the laminar organization of the most common CIN subtypes in the somatosensory cortical area of WT and *Arhgap15* ^*LacZ/LacZ*^ cortices, by immunostaining coronal sections for PVALB, CALB2, SST, and VIP. We observed a significant increase in the number of CALB2-positive neurons in bins 5, 6, and 7 of *Arhgap15* ^*LacZ/LacZ*^ mice (Figures 6A,C). Subtle changes were observed in the distribution of SST- and VIP-positive neurons in bins 10 and 6, respectively (Figures 6A,D,E). No significant alterations were observed in the distribution of PVALB-positive neurons in the absence of ARHGAP15 (Figures 6A,B). We found a slight increase in the total number of CALB2-positive CIN in *Arhgap15* ^*LacZ/LacZ*^ mice, which, however, does not reach statistical significance. The total number of PV-positive, SST-positive, and VIP-positive CINs is unaltered in the *Arhgap15* ^*LacZ/LacZ*^ mice. These results show that the stratification of adult CALB2-positive CINs in the forebrain cortex is altered in the absence of ARHGAP15, along with milder alteration in the layering of SST- and VIP-positive cells, probably due to the migratory defects observed.

### Spontaneous subclinical epileptic spikes in *Arhgap15* ^*LacZ/LacZ*^ mice

We previously observed an altered density of excitatory and inhibitory synapses on the soma of *Arhgap15* ^*LacZ/LacZ*^ cortical pyramidal neurons, and also noticed significant differences between the electroencephalography (EEG) of sleeping WT and *Arhgap15* ^*LacZ/LacZ*^ animals (Zamboni et al., 2018).

**FIGURE 7:**
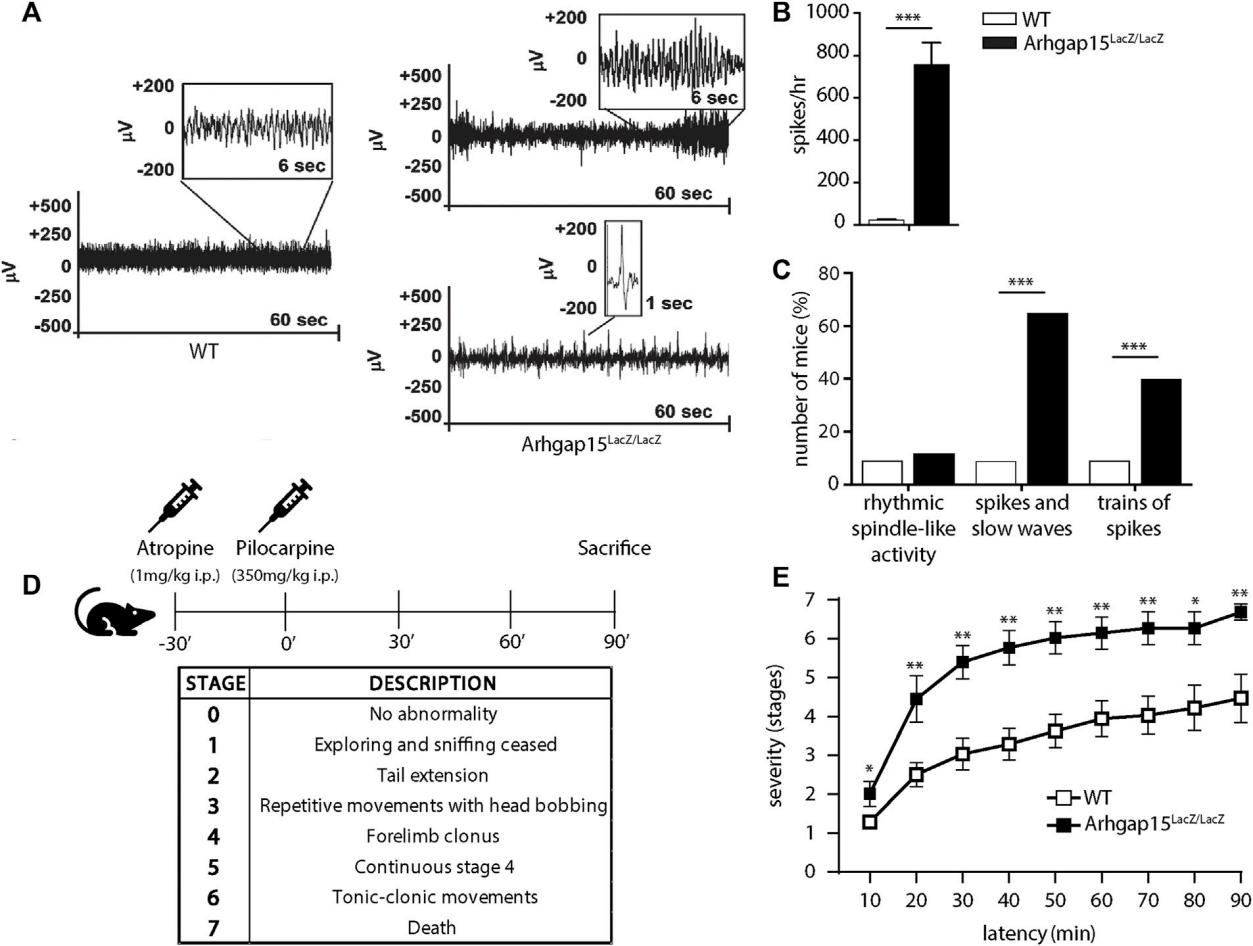
Electroencephalography and pilocarpine treatment. **(A)** Representative electroencephalograms of 1 WT and 2 *Arhgap15* ^*LacZ/LacZ*^ mice. For *Arhgap15* ^*LacZ/LacZ*^ mice, both trains of spikes (top) and a series of single spikes (bottom) are shown. **(B)** Average number of spikes per hour in WT and *Arhgap15* ^*LacZ/LacZ*^ mice traces. *p*< 0.0001. **(C)** Percentage of mice showing rhythmic spindle-like activity (left; *p* = 0.8), spikes and slow waves (center; *p* = 0.008), and trains of spikes (right; *p* = 0.002). *n* = 10 WT and 8 *Arhgap15* ^*LacZ/LacZ*^ mice. **(D)** Schematic representation of the experimental timeline (top) and a table indicating the stage classification criteria according to the modified Racine scale (bottom). **(E)** Time course of pilocarpine-induced seizure manifestation (scored as stage 1–7) of WT and *Arhgap15* ^*LacZ/LacZ*^ mice. p (from 10 to 90 min) = 0.03, 0.008, 0.004, 0.005, 0.004, 0.006, 0.008, 0.03, 0.005. *n* = 8 (5 females and 3 males) P90 mice for each genotype. Data are presented as mean ± SEM. *p*-values were calculated using unpaired two-tailed Student’s t-test **(B)**, chi-square test **(C)**, and unpaired Mann-Whitney test **(E)**. * = *p*< 0.05, ** = *p*< 0.01, *** = *p*< 0.001.

We noted that, occasionally, following moderate stress, *Arhgap15* ^*LacZ/LacZ*^ mice (5 out of 150) manifested stiffening, leg extension, and absence of movements lasting less than 5 s, which were never seen in WT mice (n > 200), probably manifestations of tonic-clonic convulsions (*i.e.*, spontaneous epileptic seizures). For this reason, we monitored and quantified the cortex-derived electrical activity of free-moving WT and *Arhgap15* ^*LacZ/LacZ*^ adult (P120) mice. The majority of mutant mice showed an abnormal EEG with epileptiform discharges, usually associated with tonic-clonic convulsions, and/or a series of single spikes, associated with less severe myoclonus (Figure 7A) (Erbayat-Altay et al., 2008). The mean number of spikes per hour was significantly greater in *Arhgap15* ^*LacZ/LacZ*^, as compared to WT mice (Figure 7B) (Purtell et al., 2018). A significantly higher fraction of *Arhgap15* ^*LacZ/LacZ*^ mice exhibited spikes and slow waves activity and/or trains of spikes, as compared to WT mice (spikes and slow waves: 1 out of 10 WT mice, 5 out of 8 KO mice; trains of spikes: 1 out of 10 WT mice, 3 out of 8 KO mice) (Figure 7C). These results indicate that *Arhgap15* ^*LacZ/LacZ*^ mice show a subclinical epileptic-like phenotype characterized by spike activity.

### Increased susceptibility to pilocarpine-induced epilepsy in *Arhgap15* ^*LacZ/LacZ*^ mice

We tested the susceptibility of *Arhgap15* ^*LacZ/LacZ*^ mice to acute treatment with pilocarpine, a widely used inducer of epilepsy (Curia et al., 2008). Mice were administered with a single dose of atropine to prevent the peripheral side effects and, after 30 min, with a single dose of pilocarpine (Figure 7D). The epileptogenic activity of the drug was assessed by scoring 7 progressive epilepsy stages, according to the modified Racine scale (Racine, 1972) (Figure 7D). *Arhgap15* ^*LacZ/LacZ*^ mice exhibited a higher likelihood to enter stages 2, 4, 5, and 6, as compared to control animals (Figure 7E). Moreover, they showed a significantly higher mortality rate at 90 min (stage 7; Figure 7E). These data confirm that the loss of ARHGAP15 increases susceptibility to drug-induced epilepsy.

### Reduced intrinsic excitability of Arhgap15^LacZ/LacZ^ CINs

**FIGURE 8:**
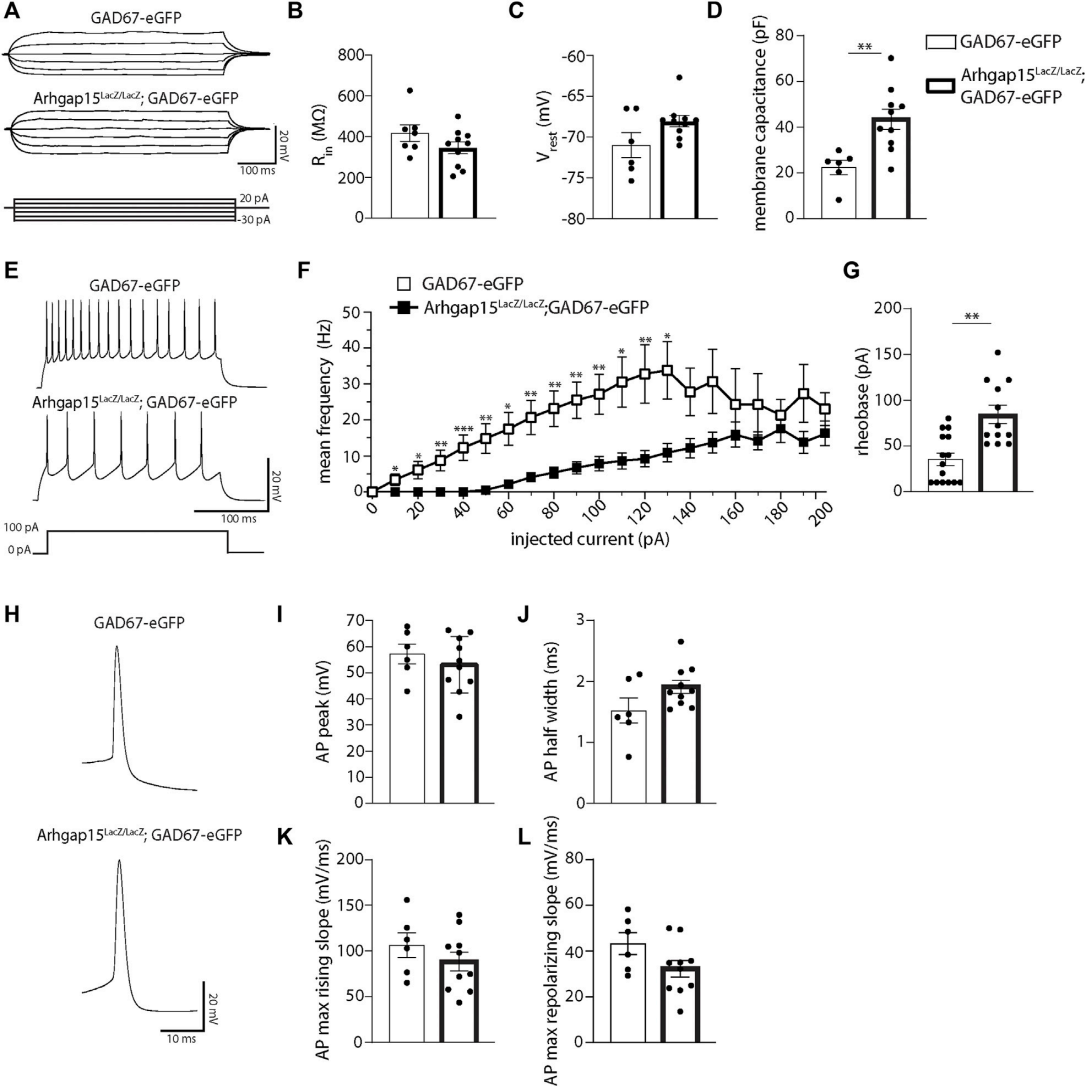
Whole-cell patch-clamp in acute slices. **(A)** Representative whole-cell current-clamp recordings of eGFP-positive CINs in acute slices prepared from adult *GAD67-eGFP* and *Arhgap15* ^*LacZ/LacZ*^ *;GAD67-eGFP* mice. Traces were obtained by injecting six current steps (from -30 to 20 pA, with 10 pA steps) lasting 500. ms. **(B–D)** Input resistance (R_in_) (B; *p* = 0.16), resting membrane potential (V_rest_) (C; *p* = 0.26), and membrane capacitance (D; p = 4 × 10^−3^) of *GAD67-eGFP* and *Arhgap15* ^*LacZ/LacZ*^ *;GAD67-eGFP* CINs. *n* = 6 cells from 4 *GAD67-eGFP* mice and 10 cells from 4 *Arhgap15* ^*LacZ/LacZ*^ *;GAD67-eGFP* mice. **(E)** Representative whole-cell current clamp recordings of action potentials (APs) evoked by 100 pA step current (bottom) for *GAD67-eGFP* (top) and *Arhgap15* ^*LacZ/LacZ*^ *;GAD67-eGFP* (middle) CINs. **(F)** Average firing frequency vs. current relationships recorded in *GAD67-eGFP* and *Arhgap15* ^*LacZ/LacZ*^ *;GAD67-eGFP* CINs in response to a set of injected current steps (from 0 to 200 pA, with 10 pA steps). p (from 0 to 200 pA) = 0.99, 0.02, 0.01, 0.005, 6 × 10^−4^, 0.006, 0.01, 0.003, 0.003, 0.002, 0.005, 0.01, 0.007, 0.01, 0.06, 0.09, 0.35, 0.27, 0.47, 0.12, 0.38. **(G)** Average rheobase (minimum amount of current required to trigger an AP) in *GAD67-eGFP* and *Arhgap15* ^*LacZ/LacZ*^ *;GAD67-eGFP* CINs. p = 2 × 10^−3^. *n* = 15 cells from 4 *GAD67-eGFP* mice and 12 cells from 4 *Arhgap15* ^*LacZ/LacZ*^ *;GAD67-eGFP* mice. **(H)** Representative APs recorded from *GAD67-eGFP* (top) and *Arhgap15* ^*LacZ/LacZ*^ *;GAD67-eGFP* (bottom) CINs. **(I–L)** Average AP peak (I; *p* = 0.45), half-width (J; *p* = 0.08), maximum rising slope (K; *p* = 0.31), and maximum repolarizing slope (L; *p* = 0.88) of evoked APs in *GAD67-eGFP* and *Arhgap15* ^*LacZ/LacZ*^ *;GAD67-eGFP* CINs. *n* = 6 cells from 4 *GAD67-eGFP* mice and 10 cells from 4 *Arhgap15* ^*LacZ/LacZ*^ *;GAD67-eGFP* mice. *p*-values were calculated using unpaired two-tailed Student’s t-test **(B,D,I−L)** and unpaired Mann–Whitney test **(C,F,G)**. Data are presented as mean ± SEM. * = *p*< 0.05, ** = *p*< 0.01, *** = *p*< 0.001.

To determine the impact of ARHGAP15 depletion on CINs electrophysiological properties, we performed whole-cell patch-clamp recordings in acute slices obtained from adult *GAD67-eGFP* and *Arhgap15* ^*LacZ/LacZ*^ *;GAD67-eGFP* mice. Interneurons showed similar input resistance (R_in_) and resting membrane voltage (V_rest_) (Figures 8A–C), whereas the membrane capacitance of *Arhgap15* ^*LacZ/LacZ*^ *;GAD67-eGFP* was significantly higher (Figure 8D). We observed a reduced firing rate of *Arhgap15*-KO CINs in response to the injection of current pulses of increasing amplitude (from 0 to 200 pA) till 130 pA of amplitude (Figures 8E,F). We also found that the rheobase was significantly higher in *Arhgap15*-KO interneurons, thus highlighting their low intrinsic excitability (Figure 8G). No differences were found in the single action potential (AP) properties (*i.e.*, AP peak amplitude, AP half-width, AP max rising slope, and AP max repolarizing slope) (Figures 8H−L).

To confirm the altered phenotype *in vitro*, we examined the firing properties of CINs in dissociated primary cultures derived from E15.5 *GAD67-eGFP* and *Arhgap15* ^*LacZ/LacZ*^ *;GAD67-eGFP* embryos at 17 DIV. Consistent with previous results, we did not observe any differences in R_in_ and V_rest_ (Supplementary Figures S2A–C). We found a decrease in the response of *Arhgap15*-KO interneurons to current injections from 20 to 180 pA (Supplementary Figures S2D,E), therefore confirming the functional defect also *in vitro*.

## Discussion

Loss of ARHGAP15 has been reported in one case of severe intellectual disability and a rare variant of Mowat–Wilson syndrome, a disease characterized by epilepsy (Smigiel et al., 2010; Mulatinho et al., 2012). Also, exome sequencing in sporadic autism spectrum disorders patients identified a synonymous *de novo* mutation in this gene (O’Roak et al., 2011). Here, we provide evidence that, in the absence of ARHGAP15, the leading process of tangentially and radially migrating CINs shows an increased tendency to deviate from the physiological trajectory. This result suggests that hyperactive RAC1 causes an abnormally fast and uncontrolled reorganization of the leading process in new directions. Strikingly, neutrophils from *Arhgap15* ^*LacZ/LacZ*^ mice also miscontrol their directionality when following a gradient of chemoattractant cytokines (Costa et al., 2011)*.* Our results show that the RAC1 negative regulator ARHGAP15 plays a similar function in migrating neurons and is required for the control of the orientation of these cells. We propose that ARHGAP15 is required to restrict the directional plasticity of the leading process and limit the rapid reorganization of the actin cytoskeleton. A similar function may be exerted by closely related RAC1 GAPs in other cell types. Hyperactive RAC1 may result in a relative instability and hyperdynamicity of the actin cytoskeleton at the tip of the leading process, resulting in an increased tendency to extend and retract the leading process, which may be interpreted as an over-physiological exploration of the environment for directional cues.

The involvement of Rho GTPases and their activating/inactivating regulators in this process is supported by the fact that RHOA, CDC42, and RAC1 participate in most intracellular events that link extracellular signaling with cytoskeletal reorganization (Hall and Lalli, 2010). Actin cytoskeleton remodeling is essential for cell migration and neurite elongation, two cellular processes that share ultrastructural features and molecular regulations (Hall and Nobes, 2000; Burridge and Wennerberg, 2004; Hua et al., 2005; Govek et al., 2011). During migration, neurons form F-Actin-rich filopodial and lamellipodial membrane protrusions at the peripheral region of the growth cone toward the direction of movement (Cooper, 2013). At the growth cone, RAC1 controls actin filaments dynamics, severing, and branching, necessary for axon elongation and turning. Consistently, in the presence of hyperactive RAC1, we previously observed defects in migration trajectory, neurite complexity, and axon guidance of other neural cell types (Zamboni et al., 2016, 2018). Although we cannot exclude RAC1-independent mechanisms, the altered migratory behavior observed in the cortex of *Arhgap15* ^*LacZ/LacZ*^ mice likely results from the hyperactivity of the RAC1-PAK1-LIMK1 transduction pathway, leading to ADF/cofilin hyperphosphorylation (hence inactivation) and aberrant actin dynamics (Liaci et al., 2021), phenomena observed in cultured *Arhgap15* ^*LacZ/LacZ*^ neurons (Zamboni et al., 2018).

The activity of other kinases downstream RAC1 has been implicated in CIN migration and neuritogenesis (*i.e.*, PAK3 and PAK6). In particular, although PAK3 expression is almost undetectable in newborn and migrating precursor interneurons, in the post-migratory MGE-derived CINs its expression increases robustly and promotes neurites outgrowth and branching after reaching the cortex (Cobos et al., 2005). Similar functions and expression dynamics have been described for PAK6 in POA-derived cells. PAK6-expressing neurons represent the post-migratory neurons derived from the POA in the late stage of maturation (Pensold et al., 2017). Interestingly, PAK3 and PAK6 are repressed during migration by DLX1/2 and DNMT1, respectively, to avoid premature differentiation of MGE-derived and POA-derived interneurons (Cobos et al., 2007; Pensold et al., 2017).

At the extracellular level, interneuron migration is controlled by a complex combination of long-range attractive and repulsive signals, short-range instructive molecules, cell-adhesion dynamics, and intrinsic motogenic factors (Métin et al., 2006). Only a few of the signals and transduction mechanisms involved have been identified, some of which belong to the family of EGF-related neuregulins (Flames et al., 2004; Alfonso et al., 2015; Zechel et al., 2016; Bartolini et al., 2017). Focusing on CIN migration in the developing cortex, RAC1 is a key hub for interpreting local cues, as it integrates and transduces several input signals into a small set of biochemical responses. RAC1 response to signals consists of an initial activation followed by a rapid return to the basal level, a process known as adaptation. Notably, *Arhgap15* ^*LacZ/LacZ*^ neutrophils show altered RAC1 activation and adaptation in response to cytokine stimulation, resulting in an altered mobilization (Campa et al., 2016), indicating that ARHGAP15 normally contributes to determining the magnitude and timescale of RAC1 activity in response to signals. Our results in neurons further support this, as *Arhgap15* ^*LacZ/LacZ*^ CINs show altered directionality and imprecise decision-making in a context of unchanged cues.

The control of CIN migration during embryonic development is critical for the establishment of their correct laminar architecture and connectivity in early postnatal life (Tamamaki et al., 1997; Clowry, 2015; le Magueresse and Monyer, 2013). During development, CINs also contribute indirectly to key aspects of forebrain organization and maturation (le Magueresse and Monyer, 2013). As a consequence of defective control of directionality, we observed a significant alteration of the CIN laminar organization in the adult cortex (*i.e.*, abnormal distribution of CALB2-positive CINs and subtle alterations in SST- and VIP-positive CINs lamination). Moreover, as we previously observed for hippocampal (Zamboni et al., 2016) and pyramidal cortical (Zamboni et al., 2018) *Arhgap15* ^*LacZ/LacZ*^ neurons, here we show that *Arhgap15* ^*LacZ/LacZ*^ CINs present an altered morphology *in vitro*. These defects, along with the observed alterations in the CINs electrophysiological properties (*i.e.*, reduced intrinsic excitability), are associated with pyramidal neuron hyperexcitability (Zamboni et al., 2016, 2018) and increased susceptibility to sporadic spontaneous and induced seizures. Notably, changes in the morphology and/or function of interneurons have been linked to neurological disorders (Juarez et al., 2022). Altered CIN development, activity, and lamination have been shown to result in an altered balance between excitation and inhibition, thereby contributing to neurological and cognitive disorders (*e.g.*, epilepsy, autism spectrum disorders, Down syndrome, Rett syndrome, and schizophrenia) (Sanacora et al., 2000; Levitt et al., 2004; Levitt, 2005; Lewis et al., 2005; Rossignol, 2011; Penzes et al., 2013). In particular, epilepsy has been causally linked to GABAergic system impairments (Tan et al., 2007). Specifically, altered CIN migration, altered inhibitory control in the postnatal brain, as well as deprivation of the neurotrophic role of GABA in early development, may result in epilepsy (Ben-Ari, 2002; Galanopoulou, 2008; Tanaka and Nakajima, 2012; Kato, 2015).

In previous work, we demonstrated that, although pyramidal cortical neurons are hyperexcitable in absence of ARHGAP15, their electrical intrinsic properties (*i.e.*, input resistance and minimum current intensity for action potential) are not altered (Zamboni et al., 2018), strongly suggesting that the defect could be about the inhibitory network. Here, we provide evidence for the reduced excitability of interneurons in the absence of ARHGAP15; this defect, together with the previously observed reduction in the number of VGAT punctae, may be the cause of the excitatory/inhibitory imbalance observed in the EEG and MEA analysis previously conducted (Zamboni et al., 2016), and of the subsequently increased susceptibility to seizure (Chen et al., 2021; Shao et al., 2019; Tai et al., 2014).

Considering that ARHGAP15 is not expressed by all CINs and it is expressed by other cells in the cortex, such as pyramidal neurons (Zamboni et al., 2018), it is expected that the overall disease phenotype entails non-cell-autonomous effects. Further experiments are needed in order to demonstrate this possible contribution. Several studies have previously shown that a specific subpopulation of CINs, the fast-spiking PVALB-positive CINs, play a central role in epilepsy (Cammarota et al., 2013; Sessolo et al., 2015); nevertheless, our present data and other studies have also shown that alterations in the SST-positive, CALB2-, and VIP-positive populations of CINs may also contribute to epileptic activity (Cobos et al., 2005). It has been shown that damage in the dendritic tree of CALB2-positive neurons may result in impaired synchronization of the entire interneuron network responsible for dendritic inhibition, resulting in an asynchronous, thus less effective, inhibition of principal cells, which may be involved in epilepsy onset (Tóth and Maglóczky, 2014). For what concerns the SST-positive neurons, studies on human and mouse models, both *in vivo* and *in vitro*, have provided evidence for a correlation between epilepsy and the loss of this CIN population (Vezzani et al., 1991; Tallent & Siggins, 1999). Also, VIP-positive neurons have been implicated in seizure susceptibility, as VIP neuropeptide is released during sustained high-frequency activity (5–40 Hz) occurring during epileptiform activity (Clynen et al., 2014). Overall, the relative contribution of each CIN subtype on the epileptic-like phenotype observed in our model remains to be fully evaluated.

## Data Availability

The raw data supporting the conclusion of this article will be made available by the authors, without undue reservation.
