# Supplementary material for: Loss of ARHGAP15 affects the directional control of migrating interneurons in the embryonic cortex and increases susceptibility to epilepsy
Source: Front Cell Dev Biol. 2022 Dec 8;10:875468. doi: 10.3389/fcell.2022.875468 (PMC9774038; doi:10.3389/fcell.2022.875468)
Supplement: Supplementary file 2 [file DataSheet1.pdf]

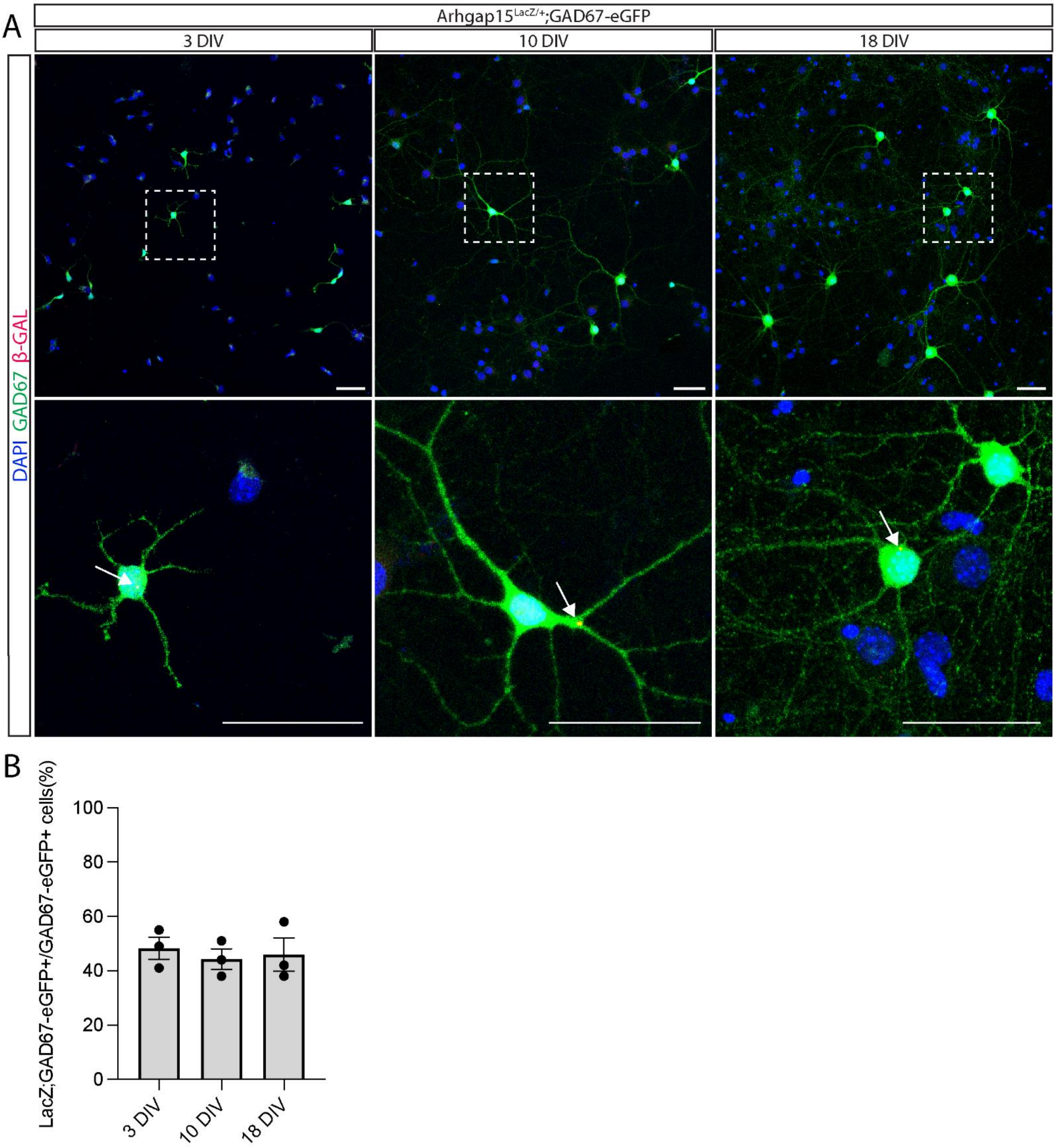

Supplementary Figure 1. β-Galactosidase expression in *Arhgap15$^{LacZ/+}$;GAD67-eGFP* cortical primary cultures.

(A) Fluorescence micrographs of cortical primary cultures derived from *Arhgap15$^{LacZ/+}$;GAD67-eGFP* embryos after 3 (left),10 (middle), and 18 (right) DIV, immunostained for β-GAL. Images on the bottom are zoomed images of the regions inside the dashed boxes. Scale bars: 20 µm. (B) Percentage of β-GAL/GAD67 double-positive cells over the total of GAD67-positive cells in cortical primary cultures after 3, 10, and 18 DIV. n=3 independent primary cultures; at least 40 (3 DIV), 30 (10 DIV), and 20 (18 DIV) neurons were analyzed for each culture. Data are presented as mean±SEM.

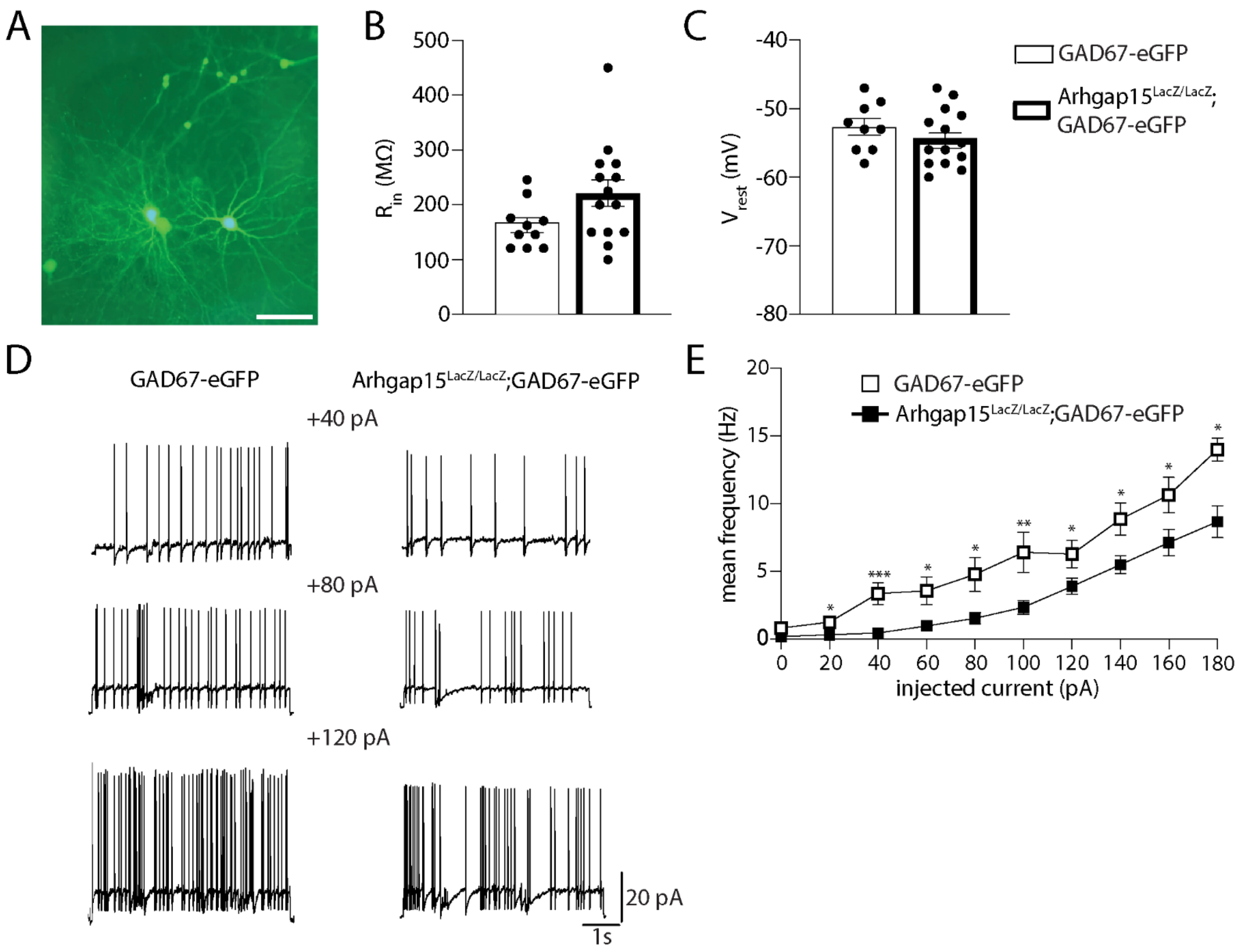

Supplementary Figure 2. In vitro current clamp analysis of *GAD67-eGFP* and *Arhgap15$^{LacZ/LacZ}$;GAD67-eGFP* primary CINs.

(A) Epifluorescence image of eGFP-positive primary neurons at 17 DIV. Scale bar: 20 µm. (B,C) Input resistance (R_{in}) (B; p=0.12) and resting membrane potential (V_{rest}) (C; p=0.34) of *GAD67-eGFP* (control) and *Arhgap15$^{LacZ/LacZ}$;GAD67-eGFP* primary CINs. (D) Representative traces of *GAD67-eGFP* (left) and *Arhgap15$^{LacZ/LacZ}$;GAD67-eGFP* (right) primary neurons at +40pA, +80pA, and +120pA pulse steps. (E) Average firing frequency vs. current relationships recorded in *GAD67-eGFP* and *Arhgap15$^{LacZ/LacZ}$;GAD67-eGFP* cultured CINs in response to a set of injected current steps (from 0 pA to 180 pA, with 20 pA steps). p(from 0 to 180 pA)=0.29, 0.02, $4x10^{-4}$, 0.01, 0.01, 0.008, 0.04, 0.02, 0.04, 0.03; n=9 *GAD67-eGFP* and 14 *Arhgap15$^{LacZ/LacZ}$;GAD67-eGFP* cells. Data are represented as mean±SEM. p values were calculated using unpaired Mann-Whitney test. *=p<0.05, **=p<0.01, ***=p<0.001.

Supplementary Video S1. Radially migrating *GAD67-eGFP* CIN. The leading process position has been tracked in blue.

Supplementary Video S2. Radially migrating *Arhgap15*$^{LacZ/LacZ}$*;GAD67-eGFP* CIN. The leading process position has been tracked in blue.
